# Development and validation of a multi-slice CTA-based prediction model for poor outcomes in isolated superior mesenteric artery dissection

**DOI:** 10.3389/fsurg.2025.1710031

**Published:** 2026-01-09

**Authors:** Kai Zhang, Huimin Hong, Zeyu Tang, Jing Feng, Qiangrong Wang, Bosheng He

**Affiliations:** 1 Department of Imaging Medicine, Second Affiliated Hospital of Nantong University, Nantong, China; 2 Department of Radiology, Dongtai People’s Hospital, Dongtai, China; 3 Department of Pathology, Affiliated Hospital of Nantong University, Nantong, China; 4 Department of Pathology, Dongtai People’s Hospital, Dongtai, China

**Keywords:** multi-slice spiral CT angiography, isolated superior mesenteric artery dissection, poor outcome, random forest model, prediction model

## Abstract

**Objective:**

A prediction model for poor outcomes in patients with isolated superior mesenteric artery dissection (ISMAD) was constructed and validated based on multi-slice spiral CT angiography (MSCTA) imaging features and clinical indicators, aiming to provide a basis for early clinical identification of high-risk patients and formulation of individualized treatment strategies.

**Methods:**

A total of 360 patients with ISMAD who were admitted to our hospital from January 2021 to December 2024 were retrospectively included. They were randomly divided into a training set (*n* = 252) and a validation set (*n* = 108) at a ratio of 7:3. The demographic characteristics, clinical symptoms and signs, laboratory test results, and MSCTA imaging features of the patients were collected. In the training set, indicators associated with poor outcomes were screened by univariate analysis, least absolute shrinkage and selection operator (LASSO) regression, and multivariate logistic regression analysis. Random forest, support vector machine, and gradient boosting models were constructed. The efficacy of the models was evaluated by the area under the receiver operating characteristic curve (AUC), the optimal model was selected, and the importance of key prediction indicators was analyzed.

**Results:**

There was no significant difference in the baseline data between the training set and the validation set (*P* > 0.05). Multivariate logistic regression analysis indicated that the visual analog scale (VAS) for abdominal pain, blood lactate levels, minimum diameter of the true lumen of the superior mesenteric artery (SMA), degree of stenosis of the SMA trunk, degree of intestinal wall thickening, and range of false lumen thrombosis formation were independent risk factors for poor outcomes (*P* < 0.05). The AUC of the random forest model (0.849) was significantly higher than that of the support vector machine (0.828) and gradient boosting models (0.818), making it the optimal model.

**Conclusion:**

A random forest model constructed based on MSCTA imaging features and clinical indicators can effectively predict poor outcomes in patients with ISMAD. Blood lactate levels, VAS score for abdominal pain, minimum true lumen diameter, degree of SMA trunk stenosis, intestinal wall thickening, and extent of false lumen thrombosis were identified as key predictors.

## Introduction

Isolated superior mesenteric artery dissection (ISMAD) is a rare acute mesenteric vascular disease. It is mainly characterized by the formation of true and false lumens after an intimal tear of the superior mesenteric artery (SMA) wall, which can lead to symptoms such as intestinal ischemia and abdominal pain. In severe cases, it can progress to intestinal necrosis and even endanger life (1). In recent years, with the popularization of multi-slice spiral CT angiography (MSCTA) technology, the detection rate of ISMAD has significantly increased (2). The outcomes of patients with ISMAD vary greatly in clinical practice. Some patients can achieve a good prognosis through conservative treatment, while others progress to adverse outcomes, such as intestinal ischemia, vascular stenosis, or occlusion, requiring emergency surgical or endovascular intervention (3). Currently, the assessment of ISMAD outcomes mainly relies on clinical experience and single imaging indicators. There is a lack of objective and accurate risk-prediction tools, making it difficult to identify high-risk patients early and develop individualized treatment plans (4). Studies have shown that the outcomes of ISMAD are closely related to the anatomical characteristics of the dissection, clinical symptoms, and inflammatory responses (5, 6). However, the predictive efficacy of single indicators is limited. Integrating MSCTA imaging features and clinical indicators to construct an efficient risk prediction model for poor outcomes remains an important research direction at present. Machine learning algorithms, with their ability to comprehensively analyze multidimensional data, have shown unique advantages in prognosis prediction in vascular diseases (7). This study aims to combine MSCTA imaging features, clinical symptoms, and laboratory indicators to construct a risk prediction model for poor outcomes of ISMAD through machine learning methods, identify key influencing factors, and provide an objective basis for clinical decision-making.

## Materials and methods

### Study subjects

A total of 360 patients (252 cases in the training set and 108 cases in the validation set) with ISMAD who were admitted to our hospital from January 2021 to December 2024 (a 4-year period) were retrospectively included. Our hospital serves as a regional referral center for vascular diseases, with an annual volume of approximately 120,000 abdominal CT angiograms, which facilitated the identification of ISMAD cases. The inclusion criteria were as follows: (1) meeting the diagnostic criteria for ISMAD (8), with the presence of SMA dissection confirmed by MSCTA and no involvement of aortic dissection; (2) being first-onset cases with complete clinical data; (3) having received at least 2 weeks of clinical follow-up with clear outcome assessment results; and (4) having complete MSCTA imaging data for extraction of relevant anatomical parameters. The exclusion criteria were as follows: (1) complications involving aortic dissection or other arterial dissection in other sites; (2) secondary SMA dissection (caused by trauma, arteritis, or tumor); (3) complications involving other critical and severe diseases, such as acute myocardial infarction or cerebral infarction; (4) a history of previous SMA surgery or interventional treatment; (5) incomplete clinical data or follow-up data; and (6) complications involving underlying diseases affecting prognosis, such as severe liver or kidney failure or malignant tumors.

### Data collection

The following information was collected through the electronic medical record system and the picture archiving and communication system.

Demographic characteristics: Age, gender, history of underlying diseases (e.g., hypertension, diabetes, and hyperlipidemia), smoking, and drinking history.

Clinical characteristics: Abdominal pain-related indicators [visual analogue scale (VAS), duration, and attack frequency], number of nausea and vomiting attacks, number of diarrhea episodes, and vital signs at admission (e.g., heart rate and systolic blood pressure).

Laboratory test indicators: Blood lactate (Lac) levels, white blood cell count (WBC), neutrophil percentage (Neu%), C-reactive protein (CRP), procalcitonin (PCT), D-dimer (DDimer), hemoglobin (Hb), albumin (Alb), alanine aminotransferase (ALT), serum creatinine (Scr), and blood glucose (Glu).

MSCTA imaging features: Diameter of the SMA trunk, minimum diameter of the true lumen of the SMA, maximum diameter of the false lumen of the SMA, ratio of true/false lumen diameter of the SMA, length of dissection involvement, degree of stenosis of the SMA trunk, number of involved branch vessels, degree of intestinal wall thickening, diameter of intestinal dilation, CT value of intestinal wall enhancement, amount of abdominal effusion, and range of false lumen thrombosis formation.

### Conservative treatment protocol

For the patients in the study who received conservative treatment, the unified protocol was implemented as follows: (1) pharmacological treatment, comprising antiplatelet therapy (aspirin 100 mg once daily) or anticoagulant therapy (warfarin, with a target international normalized ratio of 2.0–3.0, or rivaroxaban, 15 mg, twice daily), was administered based on the patient's imaging findings and clinical condition, as evaluated by the attending physician; (2) symptomatic support treatment, including analgesia, intravenous rehydration, gastrointestinal decompression, and maintenance of the water–electrolyte balance; and (3) monitoring, comprising routine monitoring of vital signs, blood lactate levels, and inflammatory indicators (e.g., CRP, PCT) during hospitalization. The duration of antiplatelet/anticoagulant therapy was at least 3 months, and the treatment plan was dynamically adjusted according to MSCTA re-examination results and clinical outcomes during follow-up.

### Outcome definition

Referencing the “Chinese Expert Consensus on the Diagnosis and Treatment of Superior Mesenteric Artery Dissection” (9) and clinical practice, and considering the follow-up characteristics of this study, patient outcomes were divided into two categories. The criteria for the poor outcome group were as follows (meeting any of the following conditions): (1) worsening of intestinal ischemia (such as aggravated abdominal pain and continuous increase in blood lactate levels) during conservative treatment, requiring surgical or interventional treatment (such as stent implantation, thrombus aspiration, or intestinal resection); (2) MSCTA re-examination indicating dissection progression (e.g., increase in dissection length ≥50% or aggravation of true lumen stenosis ≥30%); or (3) death due to ISMAD-related complications during follow-up. The criteria for the good outcome group were as follows: after conservative treatment, abdominal pain was relieved, vital signs were stable, blood lactate and inflammatory indicators returned to normal, and an MSCTA re-examination that indicated dissection stability or improvement (e.g., an increase in the scope of false lumen thrombus formation or expansion of the true lumen diameter), without the need for invasive intervention. Outcome determination was jointly confirmed by two radiologists with more than 5 years of experience in imaging diagnosis and two vascular surgeons from the Department of Vascular Surgery at our hospital (who were not listed as authors due to their specific role in outcome assessment only). When disagreements arose, consensus was reached through discussion.

### Statistical analysis

SPSS 26.0, Python 3.9.0, and R 4.3.1 software were used for statistical analysis. Measurement data conforming to the normal distribution are expressed as the mean ± standard deviation, and the independent-samples *t*-test was used for comparison between the groups. Count data are expressed as the number of cases (percentage), and the *χ*² test was used for comparison between the groups. In the training set, indicators related to poor outcomes were first screened using univariate analysis, and the variables were further compressed, and collinearity was reduced by least absolute shrinkage and selection operator (LASSO) regression. Then, a multivariate logistic regression model was used to determine the independent influencing factors, and their odds ratios and 95% confidence intervals (CIs) were calculated. Random forest, support vector machine, and gradient boosting models were constructed using Python 3.9.0 software and the scikit-learn library. Tenfold cross-validation was used to optimize the model parameters, the receiver operating characteristic (ROC) curve was plotted, and the area under the curve (AUC) value was calculated. A *P*-value < 0.05 was considered statistically significant.

## Results

### Comparison of the general data of the patients in the training and validation sets

A total of 360 patients with ISMAD were included. Of the 252 patients in the training set, 76 (30.16%) had poor outcomes, and 176 (69.84%) had good outcomes. Among those with poor outcomes, there were three deaths (3.95%), 58 patients (76.32%) received interventional therapy, and 15 (19.74%) underwent open surgery. Of the 108 patients in the validation set, 32 (29.63%) had poor outcomes, and 76 (70.37%) had good outcomes. In the poor outcome group, one patient (3.13%) died, 24 (75.00%) received interventional therapy, and seven (21.88%) underwent open surgery. There were no statistically significant differences in the general data of the patients in the training and validation sets (*P* > 0.05) (Table 1).

**Table 1:** Comparison of the general data of the patients in the training and validation sets.

Indicator	Training set (*n* = 252)	Validation set (*n* = 108)	*t*/*χ²*	*P*
Age (years)	58.62 ± 9.35	57.98 ± 8.76	0.683	0.495
Gender (male/female)	168/84	72/36	0.092	0.762
Hypertension (yes/no)	142/110	60/48	0.115	0.734
Diabetes (yes/no)	95/157	40/68	0.047	0.828
Hyperlipidemia (yes/no)	128/124	54/54	0.271	0.603
Smoking history (yes/no)	103/149	43/65	0.088	0.767
Drinking history (yes/no)	89/163	38/70	0.032	0.858
VAS score for abdominal pain (points)	5.82 ± 2.13	5.67 ± 1.98	0.651	0.516
Duration of abdominal pain (h)	18.65 ± 7.24	17.92 ± 6.89	0.915	0.361
Frequency of abdominal pain attacks (times/day)	2.85 ± 1.32	2.76 ± 1.25	0.624	0.533
Frequency of nausea and vomiting attacks (times/day)	1.92 ± 1	1.85 ± 0.98	0.612	0.540
Frequency of diarrhea (times/day)	1.36 ± 0.82	1.29 ± 0.76	0.743	0.458
Heart rate at admission (beats/min)	88.60 ± 12.31	87.90 ± 11.81	0.500	0.617
Systolic blood pressure at admission (mmHg)	136.01 ± 18.51	134.62 ± 17.93	0.997	0.319
Blood lactate (Lac, mmol/L)	1.85 ± 0.72	1.79 ± 0.68	0.824	0.410
White blood cell count (WBC, ×10⁹/L)	9.65 ± 2.38	9.48 ± 2.25	0.657	0.511
Neutrophil percentage (Neu%, %)	72.30 ± 8.51	71.81 ± 7.90	0.582	0.561
C-reactive protein (CRP, mg/L)	28.62 ± 15.31	27.91 ± 14.80	0.453	0.651
Procalcitonin (PCT, ng/mL)	0.32 ± 0.21	0.30 ± 0.19	0.897	0.370
D-dimer (D-Dimer, μg/mL)	1.25 ± 0.63	1.21 ± 0.59	0.648	0.517
Hemoglobin (Hb, g/L)	128.52 ± 15.61	127.33 ± 14.95	1.097	0.427
Albumin (Alb, g/L)	38.60 ± 4.21	38.20 ± 3.93	0.842	0.400
Alanine aminotransferase (ALT, U/L)	35.82 ± 18.65	34.99 ± 17.81	0.392	0.695
Serum creatinine (Scr, μmol/L)	86.50 ± 21.35	85.82 ± 20.71	0.279	0.780
Blood glucose (Glu, mmol/L)	6.85 ± 1.52	6.78 ± 1.45	0.472	0.637
Diameter of the main trunk of the SMA (mm)	7.25 ± 1.13	7.18 ± 1.08	0.613	0.540
Minimum diameter of the true lumen of the SMA (mm)	4.32 ± 1.25	4.25 ± 1.19	0.538	0.591
Maximum diameter of the false lumen of the SMA (mm)	5.16 ± 1.38	5.08 ± 1.32	0.574	0.566
Ratio of true/false lumen diameter of the SMA	0.84 ± 0.23	0.82 ± 0.21	0.792	0.429
Length involved in the dissection (mm)	42.60 ± 15.81	41.81 ± 15.21	0.439	0.661
Degree of stenosis of the main trunk of the SMA (%)	45.80 ± 18.61	44.91 ± 17.95	0.478	0.633
Number of involved branch vessels (branches)	1.62 ± 0.85	1.58 ± 0.81	0.487	0.626
Degree of intestinal wall thickening (mm)	3.85 ± 1.23	3.79 ± 1.18	0.562	0.574
Diameter of intestinal dilation (mm)	28.60 ± 5.30	28.10 ± 5.10	0.893	0.372
CT value of intestinal wall enhancement (HU)	58.60 ± 12.51	57.91 ± 11.80	0.487	0.626
Range of false lumen thrombosis formation (%)	38.50 ± 16.21	37.81 ± 15.61	0.374	0.708

### Univariate analysis of the factors influencing poor outcomes in isolated superior mesenteric artery dissection

In the training set, univariate analysis showed significant differences between the patients with poor outcomes and those with good outcomes in terms of VAS for abdominal pain, blood lactate levels, minimum diameter of the true lumen of the SMA, degree of stenosis of the SMA trunk, degree of intestinal wall thickening, and range of false lumen thrombosis formation (all *P* < 0.05) (Table 2).

**Table 2:** Univariate analysis of factors influencing poor outcomes in isolated superior mesenteric artery dissection.

Indicator	Poor outcome group (*n* = 76)	Good outcome group (*n* = 176)	t/*χ²*	*P*
Age (years)	57.66 ± 9.35	59.02 ± 9.30	1.064	0.289
Gender (male/female)	50/26	118/58	0.038	0.846
Hypertension (yes/no)	43/33	99/77	0.002	0.962
Diabetes (yes/no)	28/48	67/109	0.034	0.854
Hyperlipidemia (yes/no)	39/37	89/87	0.012	0.913
Smoking history (yes/no)	33/43	70/106	0.292	0.589
Drinking history (yes/no)	32/44	57/119	2.095	0.139
VAS score for abdominal pain (points)	6.71 ± 2.22	5.72 ± 2.03	3.453	0.001
Duration of abdominal pain (h)	18.85 ± 7.34	17.65 ± 7.04	1.226	0.221
Frequency of abdominal pain attacks (times/day)	2.94 ± 1.35	2.77 ± 1.02	1.096	0.273
Frequency of nausea and vomiting attacks (times/day)	1.88 ± 1.01	1.97 ± 1.15	0.591	0.555
Frequency of diarrhea (times/day)	1.46 ± 0.92	1.35 ± 0.72	0.021	0.308
Heart rate at admission (beats/min)	89.11 ± 12.33	87.65 ± 12.30	0.864	0.388
Systolic blood pressure at admission (mmHg)	136.80 ± 18.10	134.80 ± 17.50	0.824	0.411
Blood lactate (Lac, mmol/L)	1.95 ± 0.72	1.81 ± 0.32	2.140	0.033
White blood cell count (WBC, × 10⁹/L)	9.66 ± 2.38	9.64 ± 2.08	0.704	0.482
Neutrophil percentage (Neu%, %)	73.38 ± 8.60	72.10 ± 8.41	1.101	0.271
C-reactive protein (CRP, mg/L)	33.60 ± 16.7	26.2 ± 13.0	3.793	0.001
Procalcitonin (PCT, ng/mL)	0.31 ± 0.20	0.33 ± 0.25	0.617	0.538
D-dimer (D-Dimer, μg/mL)	1.27 ± 0.61	1.24 ± 0.62	0.354	0.723
Hemoglobin (Hb, g/L)	130.51 ± 15.60	126.55 ± 15.11	1.191	0.057
Albumin (Alb, g/L)	37.10 ± 4.00	38.70 ± 4.60	1.116	0.266
Alanine aminotransferase (ALT, U/L)	36.35 ± 18.60	34.47 ± 17.20	0.777	0.438
Serum creatinine (Scr, μmol/L)	87.54 ± 21.5	83.53 ± 20.50	1.404	0.162
Blood glucose (Glu, mmol/L)	6.91 ± 1.56	6.79 ± 1.48	0.581	0.561
Diameter of the main trunk of the SMA (mm)	7.36 ± 1.13	7.20 ± 1.13	1.032	0.303
Minimum diameter of the true lumen of the SMA (mm)	4.52 ± 1.3	4.20 ± 1.23	2.086	0.038
Maximum diameter of the false lumen of the SMA (mm)	5.18 ± 1.39	5.15 ± 1.32	0.163	0.871
Ratio of true/false lumen diameter of the SMA	0.85 ± 0.24	0.83 ± 0.19	0.706	0.481
Length involved in the dissection (mm)	42.91 ± 15.90	42.12 ± 15.24	0.372	0.709
Degree of stenosis of the main trunk of the SMA (%)	50.80 ± 18.80	45.10 ± 18.70	2.217	0.028
Number of involved branch vessels (branches)	1.65 ± 0.95	1.59 ± 0.75	0.536	0.592
Degree of intestinal wall thickening (mm)	4.95 ± 1.28	3.81 ± 1.21	6.744	0.001
Diameter of intestinal dilation (mm)	29.13 ± 5.40	28.41 ± 5.11	1.009	0.314
CT value of intestinal wall enhancement (HU)	57.62 ± 12.10	59.51 ± 12.17	1.134	0.258
Range of false lumen thrombosis formation (%)	40.51 ± 16.65	36.18 ± 13.81	2.143	0.033

### Multivariate logistic regression analysis of the factors influencing poor prognosis in isolated superior mesenteric artery dissection

The treatment efficacy among patients was used as the dependent variable (1 = poor prognosis group, 0 = good prognosis group) (Supplementary Table S1). The indicators with statistical significance in the univariate analysis were included in the LASSO regression for variable screening. Variables were selected using the screening criterion of lambda.1se (Supplementary Figures S1, S2). These selected variables were subsequently included in a multivariate logistic regression model. The results demonstrated that the VAS for abdominal pain, blood lactate levels, minimum diameter of the true lumen of the SMA, degree of stenosis of the SMA trunk, degree of intestinal wall thickening, and range of false lumen thrombosis formation were significantly associated with poor prognosis, and regarded as independent risk factors for poor prognosis (all *P* < 0.05) (Table 3).

**Table 3:** Multivariate logistic regression analysis of factors influencing poor outcomes in isolated superior mesenteric artery dissection.

Indicator	β	Standard error	Wald	*P*	OR	95% CI
VAS for abdominal pain	0.235	0.083	8.017	0.005	1.265	1.075–1.489
Blood lactate	1.219	0.361	11.393	0.001	3.385	1.667–6.872
CRP	0.023	0.012	3.725	0.054	1.023	1.000–1.047
Minimum diameter of the true lumen of the SMA	0.381	0.142	7.174	0.007	1.463	1.108–1.934
Degree of stenosis of the SMA trunk	0.025	0.009	7.294	0.007	1.026	1.007–1.045
Degree of intestinal wall thickening	0.634	0.144	19.239	0.001	1.884	1.420–2.500
Range of false lumen thrombosis formation	0.031	0.011	7.160	0.007	1.031	1.008–1.055

### Prediction performance of the machine learning models in the training and validation sets

**Figure 1:**
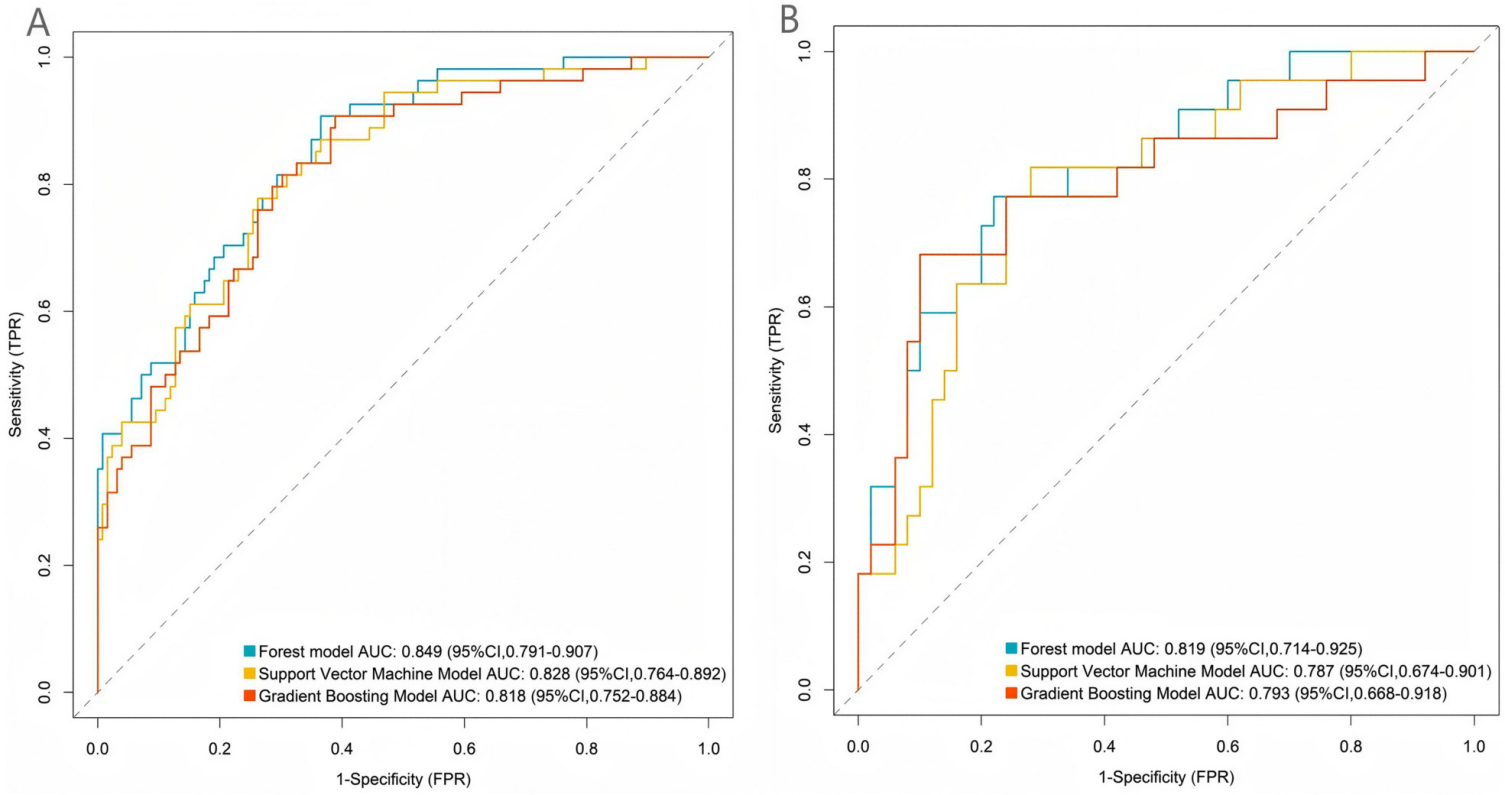
Receiver operating characteristic curves of three machine learning models for the training **(A)** and validation sets **(B)**.

Random forest, support vector machine, and gradient boosting models were used for prediction in the training and validation sets. The AUC values of the three models were 0.849, 0.828, and 0.8185, respectively. The random forest model had the largest AUC value and was thus the best model in this study (Figure 1).

### Construction of the model predicting poor prognosis in isolated superior mesenteric artery dissection

Using the random forest model, the importance of independent factors influencing poor prognosis in isolated superior mesenteric artery dissection was calculated. The importance ranking (from high to low) was as follows: blood lactate levels, degree of intestinal wall thickening, extent of thrombus formation in the false lumen, minimum diameter of the true lumen of the SMA, degree of stenosis of the SMA trunk, and VAS for abdominal pain (Supplementary Figures S3, S4).

## Discussion

ISMAD has a spontaneous onset but significant differences in outcomes. Some patients can achieve a good prognosis through conservative treatment, while high-risk patients are prone to progressing to intestinal ischemia, necrosis, and even death. Therefore, early and accurate identification of the risk of poor outcomes is crucial for formulating individualized treatment strategies (10, 11). Currently, the clinical assessment of ISMAD outcomes mostly relies on empirical judgment, lacking objective and integrated prediction tools (12). In this study, based on the imaging features of MSCTA and clinical and laboratory indicators, after variable selection by LASSO regression, multivariate analysis was used to identify that Lac levels, the minimum diameter of the true lumen of the SMA, the degree of stenosis of the SMA main trunk, the degree of intestinal wall thickening, and the range of false lumen thrombosis were independent risk factors for poor outcomes in ISMAD. A random forest prediction model was constructed, providing a quantitative basis for the early clinical identification of high-risk patients.

This study identified the abdominal pain VAS score as an independent predictor of poor outcomes in patients with ISMAD through multivariate analysis (OR = 1.265, *P* = 0.005). As the most common clinical symptom of ISMAD, the severity of abdominal pain often reflects the degree and urgency of the mesenteric ischemia. The VAS score, as a subjective yet standardized tool for pain assessment, quantifies the intensity of a patient's abdominal pain, offering strong clinical operability and reproducibility. In this study, the poor outcome group had a significantly higher abdominal pain VAS score than the good outcome group, suggesting that greater pain intensity is associated with a higher risk of progressing to intestinal ischemia, requiring surgical intervention, or death. Therefore, in clinical practice, great importance should be given to complaints of abdominal pain in patients with ISMAD, and dynamic assessment using the VAS score is recommended. In particular, for patients with persistent or worsening pain and those with high VAS scores, vigilance should be maintained for potential significant hemodynamic compromise or progression of intestinal ischemia, prompting timely imaging re-evaluation and intervention decisions to prevent adverse outcomes.

Blood lactate is a sensitive biochemical marker of tissue hypoxia. When ISMAD causes insufficient blood perfusion of the SMA, the aerobic metabolism of intestinal tissue is inhibited, anaerobic glycolysis is enhanced, and lactate production increases and is released into the blood (13). In this study, the blood lactate level in the poor outcome group was significantly higher than that in the good outcome group (*P* = 0.033), which is consistent with the previous conclusion that “elevated blood lactate during acute mesenteric ischemia indicates severe tissue hypoxia and poor prognosis” (13). Blood lactate can reflect the degree of intestinal ischemia in real time and assist in the early clinical judgment of the risk of disease progression.

The true lumen of the SMA is the main functional channel for mesenteric blood flow (14). This study showed that the minimum diameter of the true lumen of the SMA in the poor outcome group was smaller (*P* = 0.038). True lumen stenosis directly reduces effective blood perfusion, leading to ischemic injury of the intestinal mucosa and muscle layer. Accurate measurement of the true lumen diameter by MSCTA can intuitively reflect the degree of stenosis of the “functional blood flow channel,” which is closely related to the severity of intestinal ischemia and is a core imaging indicator for evaluating the prognosis of ISMAD.

The main trunk of the SMA is the “source” of the mesenteric blood supply, and the degree of its stenosis directly determines the blood flow distribution of the downstream branches (15, 16). In this study, the stenosis of the SMA main trunk in the poor outcome group was more severe (*P* = 0.028). The more significant the stenosis of the main trunk, the worse the overall mesenteric blood supply, the deeper the range and degree of the ischemic intestinal segment, and the higher the risk of intestinal necrosis and surgical intervention. The assessment of the main trunk stenosis by MSCTA provides a key basis for judging the overall blood supply status.

Intestinal wall thickening is a direct imaging manifestation of intestinal ischemic injury. In the early stages of ischemia, the intestinal wall thickens due to edema and inflammatory cell infiltration; if ischemia progresses continuously, the intestinal wall will evolve from edema to necrosis (17). In this study, the intestinal wall thickening in the poor outcome group was more significant (*P* = 0.001), indicating that the degree of intestinal wall thickening not only reflects the current severity of ischemic injury but also predicts the risk of subsequent progression to intestinal necrosis. The measurement of intestinal wall thickness with MSCTA can be used as a visual marker of the “dynamic evolution” of ischemic injury.

Moreover, the false lumen thrombosis in ISMAD compresses the true lumen through the “occupying effect,” aggravating the stenosis of the true lumen, while a large thrombus range indicates the “instability” of the dissection lesion, which is prone to false lumen expansion, rupture, or thrombus spreading to the true lumen, further disrupting hemodynamics. Thus, the assessment of the range of false lumen thrombosis helps clinicians to judge the pathological process and potential risks of the dissection (18, 19).

The random forest model constructed in this study showed significant advantages in predicting poor outcomes in ISMAD due to its multidimensional data integration. It can simultaneously incorporate MSCTA images (true lumen diameter, stenosis degree, intestinal wall thickening, and false lumen thrombosis), clinical symptoms (VAS score of abdominal pain), and laboratory indicators (blood lactate levels), and effectively handle the non-linear relationships and interactions between indicators.

Through “multi-decision-tree integration” and Bootstrap sampling, the influence of single-indicator fluctuations on the prediction results was reduced, and the prediction stability of the model for unknown data was improved. At the same time, the core prediction values of indicators such as blood lactate and intestinal wall thickening were clarified through feature importance ranking, providing a quantitative reference for the key clinical monitoring directions. The clinical translation value of this model lies in its ability to identify high-risk patients with poor outcomes at an early stage through MSCTA images combined with convenient tests, such as blood lactate levels, providing an objective basis for the decision-making between “conservative treatment vs. early interventional intervention (such as stent implantation and surgery).” Our definition of poor outcomes, based on the Chinese Expert Consensus, aligns with common international criteria, such as those used in the European Society for Vascular Surgery guidelines, which also emphasize the need for intervention in cases of worsening ischemia or dissection progression. This consistency enhances the applicability of our model in diverse clinical settings.

However, this study also had some limitations. First, it was a single-center retrospective study with a relatively limited sample size, which may have selection bias. Furthermore, our findings may not be fully generalizable to other populations due to potential differences in patient demographics, treatment protocols, and healthcare systems. Future multi-center studies are needed to validate our model across diverse settings. Second, the exploration of the mechanisms of ISMAD outcomes remained at the level of clinical and imaging associations, without in-depth exploration of the molecular biological mechanisms. Moreover, the measurement of MSCTA imaging indicators relies on manual interpretation, which may have subjective differences. In the future, AI-assisted intelligent assessment of MSCTA, using automatic segmentation and quantification technology, should be explored to further improve the model’s prediction efficiency and accuracy (20).

In conclusion, the random forest model constructed based on MSCTA images and clinical indicators in this study can effectively predict the risk of poor outcomes in ISMAD. Among them, blood lactate, the minimum diameter of the SMA true lumen, the degree of stenosis of the SMA main trunk, the degree of intestinal wall thickening, and the range of false lumen thrombosis are key influencing factors. This model provides an objective tool for the early clinical identification of high-risk patients and the formulation of individualized treatment strategies, but it still needs further verification and improvement (21).

## Data Availability

The original contributions presented in the study are included in the article/Supplementary Material; further inquiries can be directed to the corresponding author.
