# Supplementary material for: Development and validation of a multi-slice CTA-based prediction model for poor outcomes in isolated superior mesenteric artery dissection
Source: Front Surg. 2026 Jan 9;12:1710031. doi: 10.3389/fsurg.2025.1710031 (PMC12827638; doi:10.3389/fsurg.2025.1710031)
Supplement: Supplementary file 1 [file Supplementaryfile1.docx]

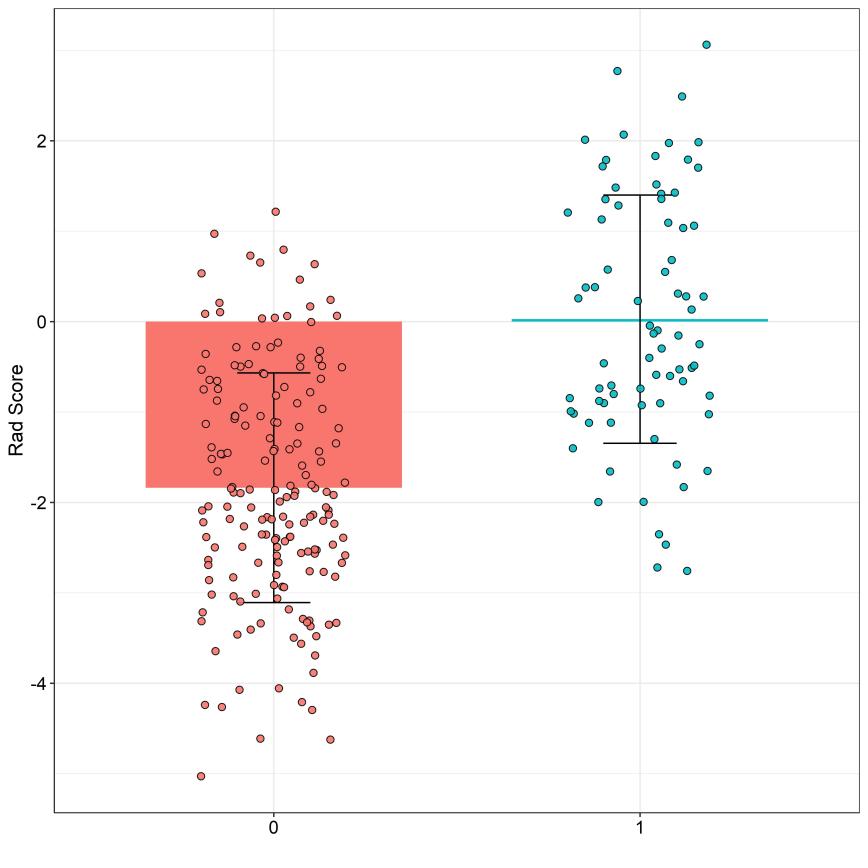


**Supplementary Figure 1.** Comparison of LASSO score differences


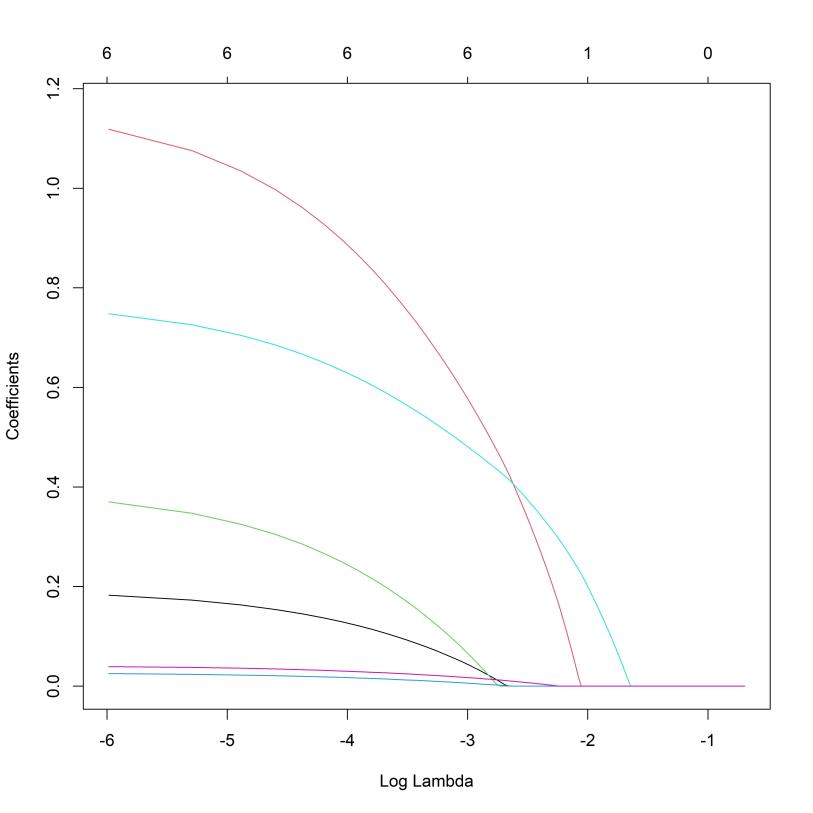


**Supplementary Figure 2.** LASSO regression analysis chart


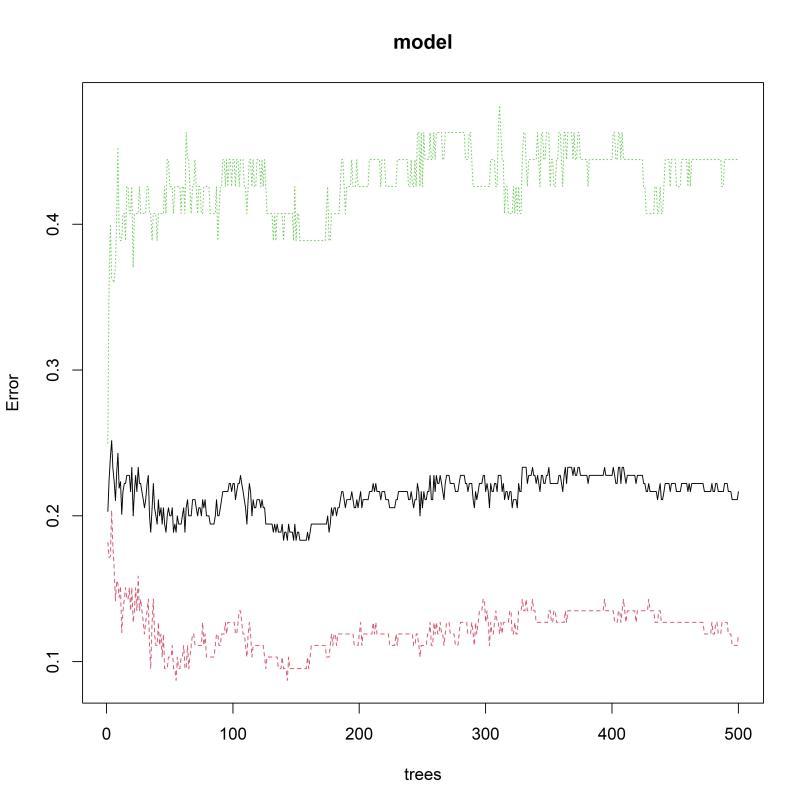


**Supplementary Figure 3.** Trend of the average out - of - bag estimation error rate with the number of decision trees

**
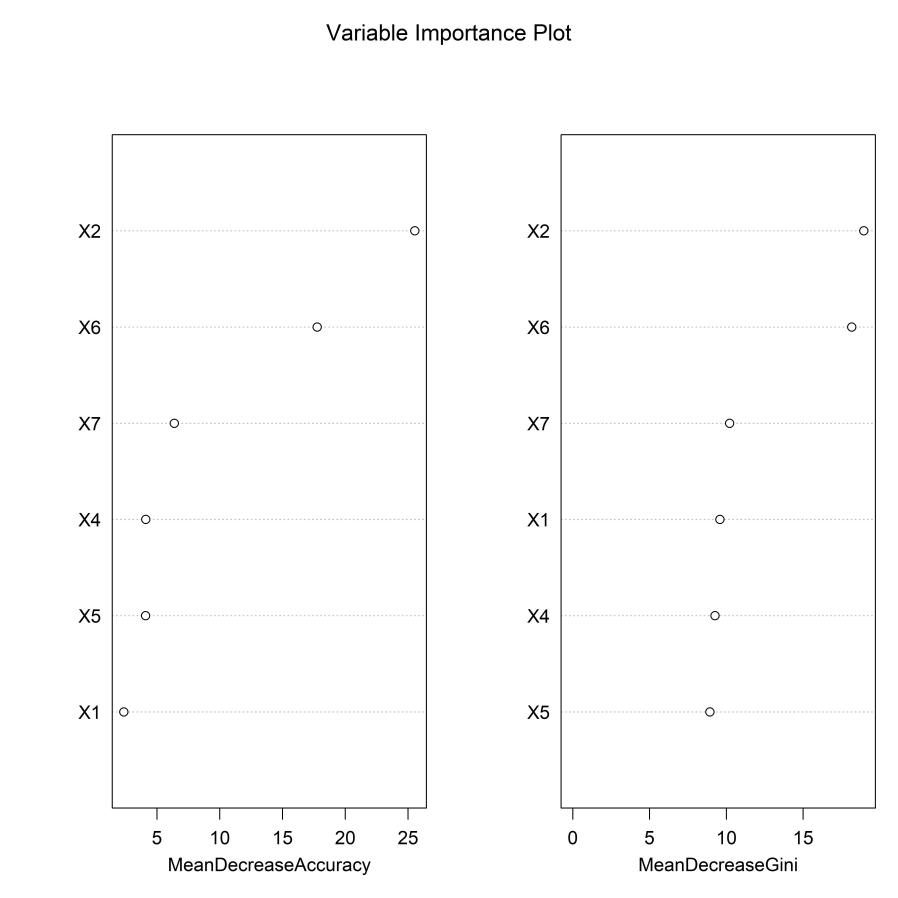
**

**Supplementary Figure 4.** Importance ranking of the random forest model
