# Supplementary material for: Development and validation of a multi-slice CTA-based prediction model for poor outcomes in isolated superior mesenteric artery dissection
Source: Front Surg. 2026 Jan 9;12:1710031. doi: 10.3389/fsurg.2025.1710031 (PMC12827638; doi:10.3389/fsurg.2025.1710031)
Supplement: Supplementary file 2 [file Table1.docx]

**Supplementary Table 1.** Variable assignment table

| Variable | Meaning | Assignment |
| --- | --- | --- |
| X1 | VAS for abdominal pain | Continuous variable |
| X2 | Blood lactate | Continuous variable |
| X3 | CRP | Continuous variable |
| X4 | Minimum diameter of the true lumen of SMA | Continuous variable |
| X5 | Degree of stenosis of the SMA trunk | Continuous variable |
| X6 | Degree of intestinal wall thickening | Continuous variable |
| X7 | Extent of thrombus formation in the false lumen | Continuous variable |
| Y | Prognosis | 1=Poor prognosis group, 0=Good prognosis group |
